# ﻿A new species of Bush frog (Anura, Rhacophoridae, *Raorchestes*) from southeastern Yunnan, China

**DOI:** 10.3897/zookeys.1151.95616

**Published:** 2023-02-28

**Authors:** Junkai Huang, Xiao Long Liu, Lingyun Du, Justin M. Bernstein, Shuo Liu, Yun Yang, Guohua Yu, Zhengjun Wu

**Affiliations:** 1 Key Laboratory of Ecology of Rare and Endangered Species and Environmental Protection (Guangxi Normal University), Ministry of Education, Guilin 541004, China; 2 Guangxi Key Laboratory of Rare and Endangered Animal Ecology, Guangxi Normal University, Guilin 541006, Guangxi, China; 3 Key Laboratory for Conserving Wildlife with Small Populations in Yunnan, Southwest Forestry University, Kunming 650224, Yunnan, China; 4 Department of Biological Sciences, Rutgers University Newark, 195 University Ave Newark, NJ 07102, USA; 5 Kunming Natural History Museum of Zoology, Kunming Institute of Zoology, Chinese Academy of Sciences, Kunming 650223, China; 6 The Management Bureau of Wenshan National Nature Reserve, Wenshan 663000, Yunnan, China

**Keywords:** *Kurixalusgryllus*, *Raorchestesgryllus*, *Raorchestesmalipoensis* sp. nov., taxonomy

## Abstract

In this study, based on morphological and molecular data, a new bush frog species is described from Yunnan, China. Eleven samples of *Raorchestesmalipoensis***sp. nov**. were collected from Malipo County, southeastern Yunnan. This species can be distinguished from other congeners by a combination of 13 morphological characters. Phylogenetic analyses based on the 16S rRNA gene indicate that these individuals form a monophyletic group, and genetic divergence between this clade and its closest relatives is higher than 3.1%, which is comparable to the divergence between recognized *Raorchestes* species. The discovery of this new species suggests that additional extensive surveys in the southeastern Yunnan would yield more amphibian lineages yet unknown to science.

## ﻿Introduction

The genus *Raorchestes* Biju, Shouche, Dubois, Dutta & Bossuyt, 2010 belongs to the family Rhacophoridae Hoffman, 1932. It includes bush frogs with adult size ranging from 10.0 mm to 50.5 mm (Priti et al. 2016). They are distinguished by the presence of a transparent/translucent vocal sac, the absence of vomerine teeth, and direct development without free swimming tadpoles (Seshadri et al. 2012). The genus *Raorchestes* currently contains 74 species, ranging from the southern tip of the Indian Peninsula to northeastern India, Indo-China, and southwestern China (Frost 2021): most are from south and Southeast Asia including southern India to Nepal, Myanmar, Thailand, Laos, southern China, Vietnam, and West Malaysia. Of the 74 recognized species, seven species have been originally described from China: *Raorchesteslongchuanensis* (Yang & Li, 1978), *R.menglaensis* (Kou, 1990), *R.andersoni* (Anderson, 1927), *R.cangyuanensis* (Wu et al., 2019), *R.dulongensis* (Wu et al., 2021), *R.hillisi* (Jiang et al., 2020), and *R.huanglianshan* (Jiang et al., 2020). Detailed ecological data is not available for the species reported in China except for *R.longchuanensis*, for which Yan et al. (2021) reported the breeding mode.

Many *Raorchestes* species from the region were described with few diagnostic characters and limited morphological data, which hampers the identification of these small-sized bush frogs (Jiang et al. 2020). In addition, the taxonomy of *Raorchestesgryllus* is under dispute. It was originally described as *Philautusgryllus* Smith, 1924, from Langbian Peaks, southern Vietnam. Biju et al. (2010) classified this species into *Raorchestes* according to the 16S sequences from Pac Ban, Tuyen Quang, northern Vietnam, and recently Poyarkov et al. (2021) suggested a transfer to *Kurixalus* based on morphological and molecular data of specimens from the type locality (Langbian, southern Vietnam).

In this work we studied specimens allocated to *Raorchestes* from Malipo County. This county is located in the southeast of Yunnan Province, and lies on the China-Vietnam border where few herpetological investigations have been conducted. During the fieldwork, we collected 11 specimens of a small-sized bush frog that could be assigned to the genus *Raorchestes* based on morphological and molecular evidence. Phylogenetically, these specimens were grouped together with a misidentified “*R.gryllus*” from Pac Ban, Tuyen Quang, northern Vietnam. However, considering that the type locality of *Philautusgryllus*, Langbian Plateau, is 1200 km far from the China-Vietnam border and that obvious morphological differences exist between *Philautusgryllus* and the lineage consisting of individuals from China-Vietnam border region, we consider that these specimens represent a new species that we formally describe here.

## ﻿Materials and methods

### ﻿Sampling

**Figure 1.**
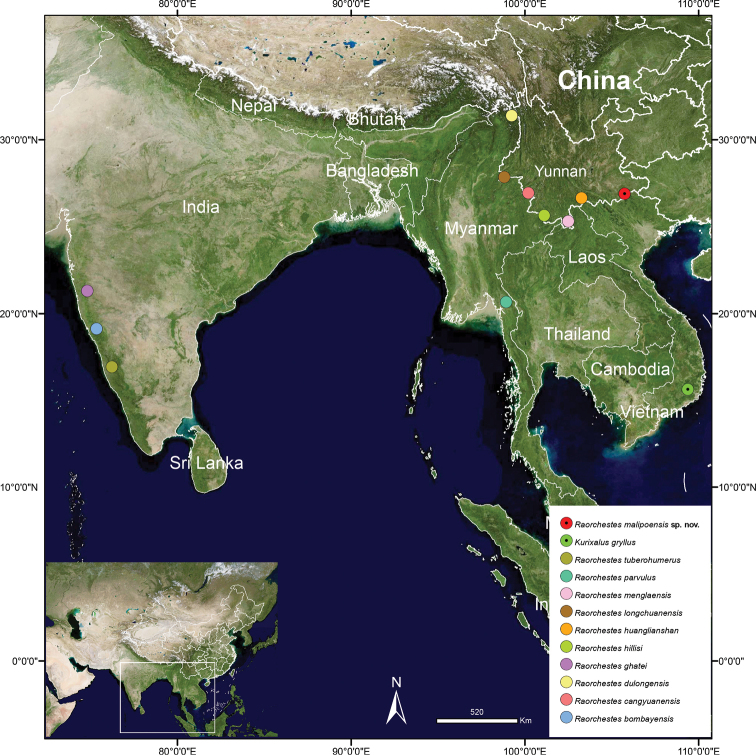
The type locality of *Raorchestesmalipoensis* sp. nov., its closest relatives, and also *Kurixalusgryllus* (previously *Raorchestesgryllus*).

**Figure 2.**
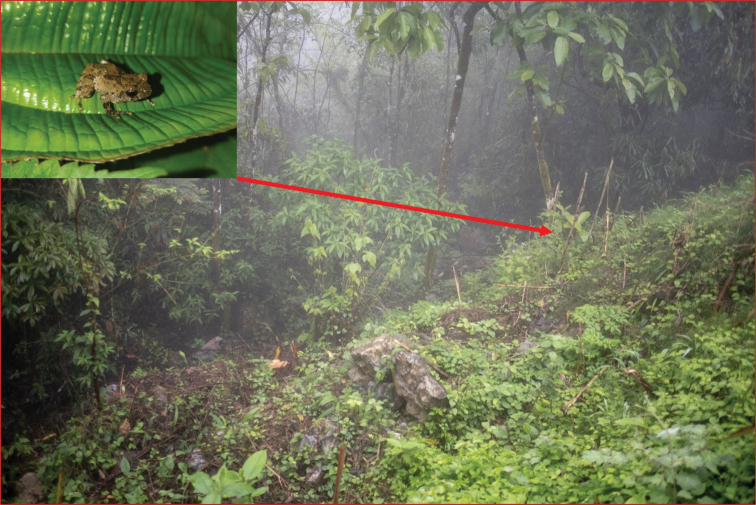
Habitat at the type locality of *Raorchestesmalipoensis* sp. nov., Malipo County, Yunnan Province, 23.182°N, 104.78°E, elevation 1496 m, China.

Fieldwork was conducted at Malipo County, Yunnan Province, China (23.182°N, 104.78°E, elevation 1496 m). Six specimens were collected on 7 May 2019 (Figs 1, 2) and another five specimens were collected on 22 July 2020. Specimens were collected by hand and subsequently euthanized with 20% ethanol following standard euthanasia protocols for amphibians. Liver or muscle tissues were taken from the specimens and preserved in 95% ethanol before fixing them in 75% ethanol. Voucher specimens SWFU 3110, SWFU 3113, SWFU 3114, SWFU 3116, SWFU 3111, and SWFU 3112 were deposited at Southwest Forestry University (**SWFU**). GXNU 000338, GXNU 000339, GXNU 000340, GXNU 000341, GXNU 000342 were deposited at Guangxi Normal University (**GXNU**).

### ﻿Morphology and morphometrics

All the measurements were made with slide calipers to the nearest 0.1 mm. Morphological terminology and measurement methods followed Fei et al. (2009). The morphological characters include: snout-vent length (**SVL**); head length (**HL**); head width (**HW**); snout length (**SL**); internarial distance (**INS**); interorbital distance (**IOS**); eye horizontal diameter (**EHD**); maximum width of upper eyelid (**UEW**); tympanum diameter (**TD**); forelimb and hand length (**FAHL**); width of lower arm (**LAW**); hand length (**HAL**); femur length (**FML**); tibia length (**TBL**); length of tarsus and foot (**TFL**); foot length (**FOL**); tibia width (**TBW**); and femur width (**FMW**). Morphological measurements of the specimens are given in Table 1. Males and females (breeding individuals) were identified based on the presence or absence of an external single subgular vocal sac. Comparative morphological data of congeneric species were taken from previous studies and are presented in Table 2.

**Table 1.** Measurements (mm) of adult specimens in the type series of *Raorchestesmalipoensis* sp. nov. Abbreviations defined in the Materials and methods.

Sex	Males (n = 4)				Females (n = 2)		Males (n = 3)			Females (n = 2)	
Catalog No.	SWFU 3110	SWFU 3113	SWFU 3114	SWFU 3116	SWFU 3111	SWFU 3112	GXNU 000338	GXNU 000339	GXNU 000341	GXNU 000340	GXNU 000342
SVL	17.1	17.0	16.5	14.7	19.3	19.0	17.5	17.7	17.3	18.7	18.3
HL	5.7	5.8	6.3	5.2	6.5	7.9	5.9	6.4	5.7	6.3	6.7
HW	7.7	7.5	8.2	5.5	8.2	7.9	6.5	6.8	6.4	7	7
SL	2.5	2.6	1.8	2.5	2.9	2.6	2	2.6	2.4	2.5	2.2
INS	1.5	2.2	2.2	2.0	2.2	2.1	2.2	2.1	2.3	2.2	2
IOS	2.7	2.7	2.9	2.9	3.2	2.9	2.9	2.6	2.9	2.7	2.9
UEW	1.2	1.2	1.9	1.4	1.6	1.7	1.6	1.3	1.4	1.4	1.7
EHD	2.2	2.1	2.1	2.6	2.6	2.8	2.4	2.6	2.4	2.5	2.2
TD	1.4	1.4	1.5	1.3	1.1	1.5	1.2	1.5	1.4	1.6	1.4
FAHL	9.3	9.3	8.8	7.0	8.6	9.6	7.8	8.2	7.2	7.3	7.1
HAL	5.6	5.6	5.3	4.2	5.2	5.6	5.3	5.2	5.3	4.6	5.4
LAW	1.7	2.0	1.6	1.3	1.4	1.6	1.5	1.2	1.6	1.3	1.3
TBL	9.1	8.7	8.4	7.5	9.1	9.2	9	8.8	8	8.7	8.8
FML	7.5	9.2	8.3	7.1	8.0	10.2	7.5	8	7.5	8.2	7.9
TBW	2.1	2.6	1.8	1.5	2.0	2.8	1.7	1.7	1.8	1.8	1.9
TFL	9.8	10.9	10.6	8.8	10.0	11.8	10.8	11	10.1	9.6	10.7

**Table 2.** The source of morphological data for *Raorchestes* species used in this study.

ID	*Raorchestes* species	Literature
3	*Raorchestesghatei* Padhye, Sayyed, Jadhav, & Dahanukar, 2013	Padhye et al. 2013
4	*Raorchestesparvulus* (Boulenger, 1893)	Bossuyt and Dubois 2001
5	*Raorchestescangyuanensis* Wu, Suwannapoom, Xu, Murphy, & Che, 2019	Wu et al. 2019
6	*Raorchesteslongchuanensis* (Yang & Li, 1978)	Al-Razi et al. 2020 b; Yang and Li 1978
7	*Raorchestesmenglaensis* (Kou, 1990)	Jiang et al. 2020
8	*Raorchesteshillisi* Jiang Ren, Guo, Wang & Li, 2020	Jiang et al. 2020
9	*Raorchesteshuanglianshan* Jiang, Wang, Ren, & Li, 2020	Jiang et al. 2020
10	*Raorchestesdulongensis* Wu, Liu, Gao, Wang, Li, Zhou, Yuan, & Che, 2021	Wu et al. 2021
11	*Raorchestesandersoni* (Ahl, 1927)	Bossuyt and Dubois 2001
12	*Raorchestesrezakhani* Al-Razi, Maria, & Muzaffar, 2020	Al-Razi et al. 2020 a
13	*Raorchestesannandalii* (Boulenger, 1906)	Che et al. 2020

### ﻿DNA sequencing and analyses of sequences

Total DNA was extracted using a commercial tissue DNA isolation kit (Chenlu Biotech, China). For seven specimens in this study, the mitochondrial gene 16S ribosomal RNA (16S rRNA) gene was sequenced. The fragments of 16S rRNA were amplified using primers 16Sar-L (5’–CGCCTGTTTATCAAAAACAT–3’) and 16Sbr-H (5’–CCGGTCTGAACTCAGATCACGT–3’) (Palumbi et al. 1991). Polymerase chain reactions (PCR) amplifications were performed in a 25 μl reaction volume with an initial denaturation at 94 °C for 5 min, followed by 35 cycles of 94 °C for 1 min, 51 °C for 1 min, 72 °C for 1 min, and a final extension at 72 °C for 10 min. The PCR products were sequenced using an ABI 3730 automated sequencer. To study the phylogenetic relationships among *Raorchestes* species, matrilineal genealogies were reconstructed based on the 16S fragment. Fifty-two sequences of *Raorchestes* and representative outgroups (Jiang et al. 2020) were downloaded from GenBank (Table 3). The dataset was checked by eye and manually adjusted using MEGA 6.0 with default settings (Tamura et al. 2013), and the alignment was checked by eye and adjusted manually. JMODELTEST v. 2.1.7 (Darriba et al. 2012) was used to select an appropriate nucleotide substitution model for Bayesian Inference (**BI**). The GTR+G+I model was chosen as the best-fit model following the Bayesian information criterion (BIC; Posada 2008). Bayesian analysis was performed using MrBayes 3.2 (Ronquist et al. 2012). For BI analyses, the Monte Carlo Markov chain length was run for 120,000,000 generations and sampled every 100 generations with a burn-in of 25%. Convergence was assessed by the average standard deviation of split frequencies (below 0.01) and ESS values (greater than or equal to 200) in TRACER 1.5 (Rambaut and Drummond. 2009). Maximum likelihood (**ML**) analyses were performed using RAxML v. 8.2.10 (Stamatakis 2014) with 1000 rapid bootstrap replicates under GTR+I+G nucleotide substitution model for the concatenated dataset (Stamatakis, 2014). Mean genetic distances (uncorrected p-distance) between and within species were calculated in MEGA v. 6.0.6 (Tamura et al. 2013) based on 16S sequences.

**Table 3.** Information on voucher numbers, GenBank accession numbers, and localities of specimens used in this study; for collections and their abbreviations see Material and methods.

Species	Voucher No.	GenBank No.	Locality	Resource
**Ingroup**				
*Raorchestesmalipoensis* sp. nov.	SWFU 3110	ON128247	Malipo, Yunnan, China	This study
*Raorchestesmalipoensis* sp. nov.	SWFU 3111	ON128241	Malipo, Yunnan, China	This study
*Raorchestesmalipoensis* sp. nov.	ROM 30288	GQ285674	Pac Ban, Tuyen Quang, Vietnam	Li et al. 2009
*Raorchestesmalipoensis* sp. nov.	GXNU 000338	ON128246	Malipo, Yunnan, China	This study
*Raorchestesmalipoensis* sp. nov.	GXNU 000339	ON128245	Malipo, Yunnan, China	This study
*Raorchestesmalipoensis* sp. nov.	GXNU 000340	ON128244	Malipo, Yunnan, China	This study
*Raorchestesmalipoensis* sp. nov.	GXNU 000341	ON128243	Malipo, Yunnan, China	This study
*Raorchestesmalipoensis* sp. nov.	GXNU 000342	ON128242	Malipo, Yunnan, China	This study
*Raorchestesdulongensis*	KIZ 035082	MW537814	Qinlangdang, Yunnan, China	Wu et al. 2021
*Raorchesteshillisi*	CIB 116331	MT488411	Xiding, Yunnan, China	Jiang et al. 2020
*Raorchesteslongchuanensis*	KIZ 048468	MN475870	Unknown	Wu et al. 2019
*Raorchestesparvulus*	LSUHC:11118	MH590201	Gunung Stong, Kelantan, Malaysia	Chan et al. 2018
*Raorchestesmenglaensis*	CIB 116349	MT488410	Menglun, Yunnan, China	Jiang et al. 2020
*Raorchesteshuanglianshan*	CIB 116365	MT488414	Lvchun, Yunnan, China	Jiang et al. 2020
*Raorchestescangyuanensis*	KIZ 015855	MN475866	Cangyuanensis, Yunnan, China	Wu et al. 2019
*Raorchestestuberohumerus*	CESF 148	JX092697	Western Ghats, India	Vijayakumar et al. 2014
*Raorchestesbombayensis*	CESF 1010	JX092657	Western Ghats, India	Vijayakumar et al. 2014
*Raorchestesghatei*	AGCZRL Amphibia 128	KF366391	Western Ghats, India	Padhye et al. 2013
*Raorchestesgriet*	CESF 073	JX092654	Western Ghats, India	Vijayakumar et al. 2014
*Raorchestescoonoorensis*	CESF 439	JX092716	Western Ghats, India	Vijayakumar et al. 2014
*Raorchestescharius*	CESF 132	JX092691	Western Ghats, India	Vijayakumar et al. 2014
*Raorchestesmarki*	CESF 467	JX092719	Western Ghats, India	Vijayakumar et al. 2014
*Raorchestesindigo*	CESF 138	KM596557	Kudremukh Massif, Western Ghats, India	Vijayakumar et al. 2014
*Raorchestesemeraldi*	CESF 1365	KM596556	Valparai plateau, Western Ghats, India	Vijayakumar et al. 2014
*Raorchestesponmudi*	CESF 063	JX092651	Western Ghats, India	Vijayakumar et al. 2014
*Raorchestesaureus*	CESF 1164	KM596540	Malabar, Western Ghats, India	Vijayakumar et al. 2014
*Raorchestesmontanus*	CESF 130	KM596552	Western Ghats, India	Vijayakumar et al. 2014
*Raorchestestinniens*	CESF 438	JX092715	Western Ghats, India	Vijayakumar et al. 2014
*Raorchestesprimarrumfi*	CESF 442	KM596575	Nilgiri Massif, Western Ghats,India	Vijayakumar et al. 2014
*Raorchestessignatus*	Unknow	AY141841	Sri Lanka	Meegaskumbura et al. 2002
*Raorchesteschromasynchysi*	CESF 1127	JX092667	Western Ghats, India	Vijayakumar et al. 2014
*Raorchesteschotta*	CESF 1003	JX092656	Western Ghats, India	Vijayakumar et al. 2014
*Raorchestesnerostagona*	CESF 1061	JX092661	Western Ghats, India	Vijayakumar et al. 2014
*Raorchesteskadalarensis*	CESF 1766	JX092701	Western Ghats, India	Vijayakumar et al. 2014
*Raorchestesagasthyaensis*	CESF 492	JX092723	Western Ghats, India	Vijayakumar et al. 2014
*Raorchestestravancoricus*	CESF 473	JX092721	Western Ghats, India	Vijayakumar et al. 2014
*Raorchestesluteolus*	CESF 1012	JX092659	Western Ghats, India	Vijayakumar et al. 2014
*Raorchestesbeddomii*	CESF 072	JX092653	Western Ghats, India	Vijayakumar et al. 2014
*Raorchestestheuerkaufi*	CESF 1342	JX092693	Western Ghats, India	Vijayakumar et al. 2014
*Raorchestesmunnarensis*	CESF 094	JX092655	Western Ghats, India	Vijayakumar et al. 2014
*Raorchestesanili*	CESF 386	JX092708	Western Ghats, India	Vijayakumar et al. 2014
*Raorchestesresplendens*	CESF 1258	JX092683	Western Ghats, India	Vijayakumar et al. 2014
*Raorchestesdubois*	CESF 114	JX092668	Western Ghats, India	Vijayakumar et al. 2014
*Raorchesteskakachi*	CESF 1385	KM596558	Western Ghats, India	Vijayakumar et al. 2014
*Raorchesteskaikatti*	CESF 444	JX092718	Western Ghats, India	Vijayakumar et al. 2014
*Raorchestessushili*	CESF 1259	JX092684	Western Ghats, India	Vijayakumar et al. 2014
*Raorchestesflaviocularis*	CESF 1252	KM596549	Manalar Plateau, Western Ghats, India	Vijayakumar et al. 2014
*Raorchestesochlandrae*	CESF 1111	JX092666	Western Ghats, India	Vijayakumar et al. 2014
*Raorchestesmanohari*	CESF 1187	JX092674	Western Ghats, India	Vijayakumar et al. 2014
*Raorchestesuthamani*	CESF 483	JX092722	Western Ghats, India	Vijayakumar et al. 2014
*Raorchesteschlorosomma*	FB-2008c	EU450017	Munnar, Idukki, Kerala, India	Biju and Bossuyt 2009
*Raorchestescrustai*	CESF 1199	JX092677	Western Ghats, India	Vijayakumar et al. 2014
*Raorchestesgraminirupes*	CESF 044	JX092649	Western Ghats, India	Vijayakumar et al. 2014
*Raorchestesjohnceei*	CESF 1236	JX092679	Western Ghats, India	Vijayakumar et al. 2014
*Raorchestesglandulosus*	CESF 1080	JX092665	Western Ghats, India	Vijayakumar et al. 2014
*Raorchestesjayarami*	CESF 1260	JX092686	Western Ghats, India	Vijayakumar et al. 2014
*Raorchestesbobingeri*	CESF 1238	JX092680	Western Ghats, India	Vijayakumar et al. 2014
*Raorchestesakroparallagi*	CESF 061	JX092650	Western Ghats, India	Vijayakumar et al. 2014
**Outgroup**				
*Philautusabditus*	ROM 33145	GQ285673	Krong Pa, Gia Lai, Vietnam	Li et al. 2009

## ﻿Results

**Figure 3.**
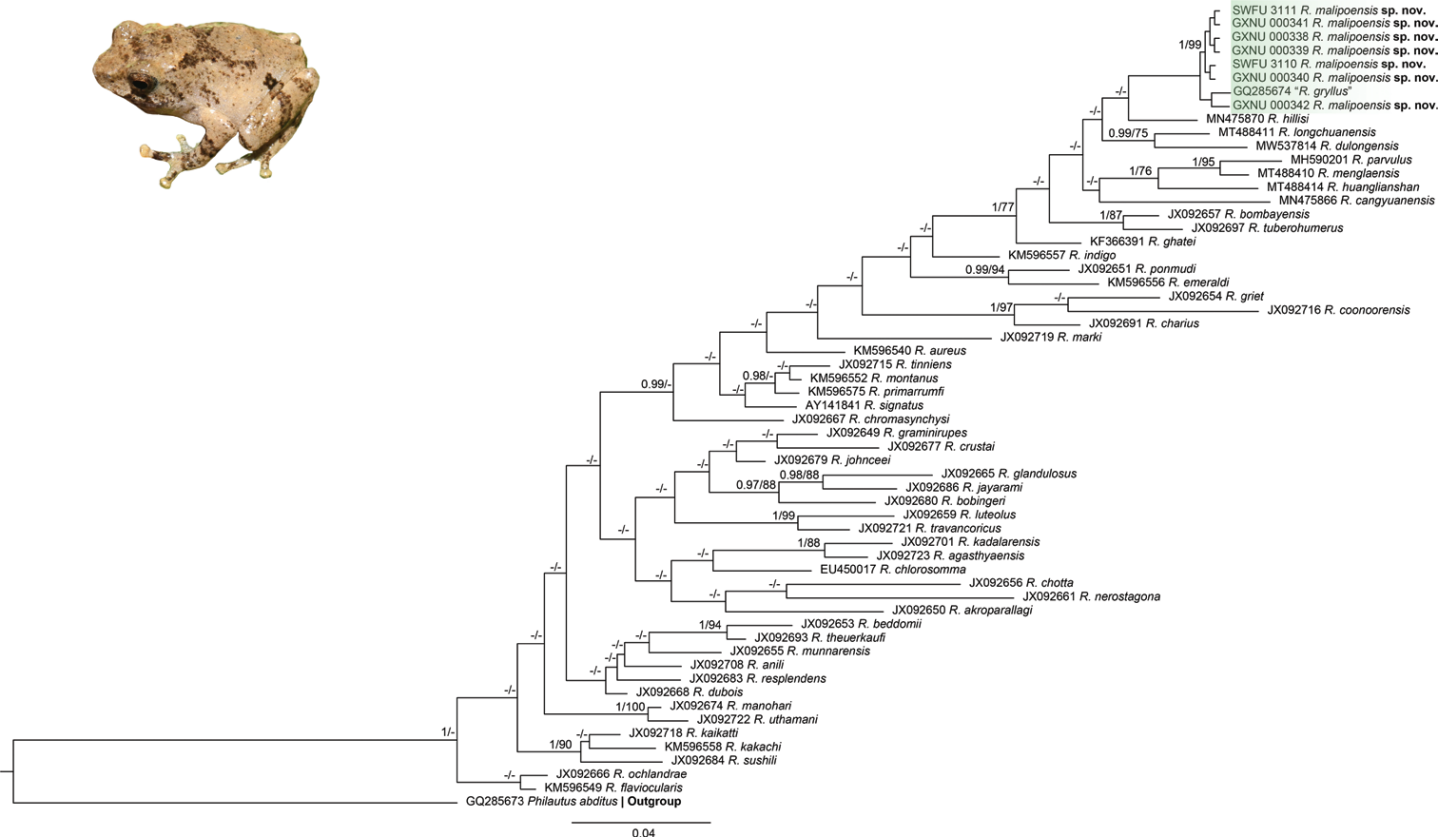
Phylogram of *Raorchestes* derived from analyses of concatenated DNA fragments of the mitochondrial 16S rRNA. Nodal support values with Bayesian posterior probability (BPP) > 0.95 / ML inferences (ML-BS) > 70 are shown near the respective nodes. A “-” denotes a Bayesian posterior probability < 0.95 and bootstrap support < 70. The scale bar represents 0.1 nucleotide substitutions per site.

The final DNA sequence dataset is consisted of 59 sequences and the length of the sequence alignment is 542 base pairs (bp) (Table 3), of which 194 sites are variable and 135 are parsimony informative. The BI and ML trees had almost identical topologies (Fig. 3). The samples from Malipo County, Yunnan Province form a monophyletic group and the sample from Pac Ban, Tuyen Quang (northern Vietnam) previously identified as *R.gryllus* was also nested in the clade with strong support (Fig. 3). Genetic distances between the samples from Malipo County and the other species of *Raorchestes* varied from 3.1% (*R.longchuanensis*) to 6.0% (*R.huanglianshan*) (Table 4).

**Table 4.** Uncorrected pairwise sequence divergence (%) among 16S ribosomal RNA mtDNA sequences, including *R.malipoensis* sp. nov., *R.dulongensis*, *R.hillisi*, *R.longchuanensis*, *R.menglaensis*, *R.huanglianshan*, *R.cangyuanensis*, *R.parvulus*, *R.bombayensis*, *R.tuberohumerus*, and *R.ghatei* as shown in phylogenetic tree presented in Fig. 3.

Species	1	2	3	4	5	6	7	8	9	10
*R.malipoensis* sp. nov.										
*R.bombayensis*	4.5									
*R.tuberohumerus*	4.8	2.0								
*R.ghatei*	4.5	4.2	4.0							
*R.parvulus*	5.9	5.1	5.9	5.4						
*R.cangyuanensis*	5.7	6.6	6.8	5.6	7.1					
*R.longchuanensis*	3.1	4.7	4.9	4.0	4.0	5.6				
*R.menglaensis*	5.2	4.2	4.9	5.7	2.0	5.9	4.7			
*R.hillisi*	4.3	4.9	5.2	4.3	61	6.3	4.2	5.4		
*R.huanglianshan*	6.0	6.1	6.4	5.2	45	6.8	5.4	4.5	5.4	
*R.dulongensis*	5.2	6.1	6.4	5.0	6.6	6.6	4.2	6.2	3.3	5.7

### ﻿Taxonomic account

#### ﻿ Raorchestes malipoensis sp. nov.

Taxon classificationAnimaliaAnuraRhacophoridae

7FB90D68-4653-5656-A75F-8A7A654368E5

https://zoobank.org/0DCF253A-45E1-4354-9C6B-AA44E7C6C309

Fig. 4 , Table 1

“Pseudophilautusgryllus” (Li et al. 2009).
“Raorchestesgryllus” (Biju et al. 2010).

##### Holotype.

GXNU 000339, adult male, collected from Malipo County, Yunnan Province (23.182°N, 104.78°E, elevation 1496 m) on 22 July 2020 by Shuo Liu.

##### Paratypes.

SWFU 3110, SWFU 3113, SWFU 3114, SWFU 3116, GXNU 000338, GXNU 000341 (six adult males), SWFU 3111, SWFU 3112, GXNU 000340, GXNU 000342 (four adult females), collected at the same locality as the holotype on 22 July 2020 by Xiaolong Liu and Shuo Liu.

##### Diagnosis.

The genus *Raorchestes* is a group of small frogs, diagnosed primarily on the basis of an adult snout-vent length between 15 and 45 mm; vomerine teeth absent; large gular pouch transparent while calling; nocturnally active; direct development without free-swimming tadpoles in all species for which the development is known (Biju et al. 2010). Although the mode of development in the new species remains unknown, *R.malipoensis* sp. nov. is placed in the genus *Raorchestes* due to the combination of following characters: small body size, vomerine teeth absent, single translucent external subgular vocal sac present, and tips of all fingers and toes expanded into discs with circum-marginal grooves. The new species is distinguished from geographically and molecularly relevant congeners by the following combination of characters: (1) very small body size (males SVL 14.6–17.7 mm, *n* = 7; females SVL 18.3–19.3 mm, *n* = 4); (2) head wider than long; (3) tympanum small, supratympanic fold distinct; (4) tips of all fingers and toes yellow; (5) webbing formula (I 2 – 2 II 2 – 2 III 2 – 3 IV 3 – 2 V); (6) inner and outer metacarpal tubercle indistinct; (7) heels not meeting when limbs held at right angles to body; (8) tibiotarsal articulation reaching anterior border of eye when hindlimb is stretched alongside of body; (9) iris golden brown; (10) nuptial pad small and milky white; (11) inner metatarsal tubercle rounded, outer metatarsal tubercle absent; (12) fingers and toes having lateral dermal fringe; and (13) interorbital distance larger than eye horizontal diameter.

##### Description of the holotype.

**Figure 4.**
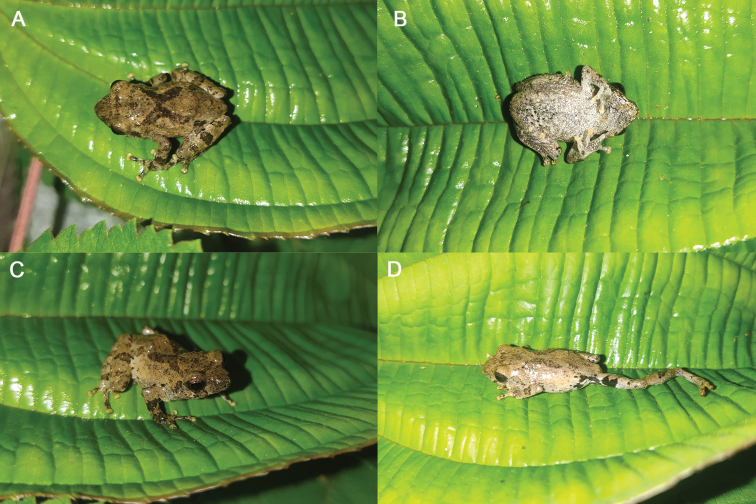
Holotype (GXNU 000339) of *Raorchestesmalipoensis* sp. nov. in life.

Adult male (Fig. 4), body size small (SVL 17.7 mm); head wider than long (HL 6.4 mm; HW 6.8 mm); top of head relatively flat; snout rounded in profile, projecting beyond lower jaw; snout length almost equal to interorbital distance at narrowest point (SL 2.6 mm; IOS 2.6 mm); the canthus rostralis rounded, loreal region slightly concave; tympanum small (TD 1.5 mm); internarial distance wider than maximum width of upper eyelid (INS 2.1 mm; UEW 1.3 mm); nostril slightly closer to tip of snout than to anterior corner of eyes; tongue pyriform, with a deep notch at posterior tip; vomerine teeth absent; pineal ocellus absent; eyes moderately large (EHD 2.6 mm) and protruding, pupil horizontal; supratympanic fold distinct, from posterior corner of eye to above insertion of arm.

Forelimbs fairly robust (FAHL 8.2 mm); relative finger lengths: I < II < IV < III, tips of all four fingers expanded into discs with circum-marginal grooves; all fingers with lateral dermal fringes on both sides; subarticular tubercles distinct, rounded; supernumerary tubercles absent; no webbing between fingers; inner and outer metacarpal tubercle indistinct; nuptial pad is small and milky white on dorsal surface of the first finger.

Foot long and relatively robust (TFL 11 mm), longer than tibia length (TBL 8.8 mm); relative toe lengths: I < II < V < III < IV; tips of toes with discs having circum-marginal grooves, toe discs smaller than finger discs; all toes with lateral dermal fringes on both sides; subarticular tubercles distinct, rounded; supernumerary tubercles absent; webbing formula (I 2 – 2 II 2 – 2 III 2 – 3 IV 3 – 2 V); inner metatarsal tubercle rounded, outer metatarsal tubercle absent.

Dorsal surfaces of head, body, forelimbs, thighs, and tibia rough with small granules; upper eyelid with several small granules; throat, chest, and ventral surfaces of forelimbs smooth; abdomen, ventral side of thigh, and area around vent with granules; dorsolateral folds absent.

##### Coloration of holotype in life.

For coloration of the holotype in life see Fig. 4. Dorsal surface beige, with pale brown band between eyes; dorsal surface with a dark brown X-shaped marking; pale brown interorbital rectangle between eyes; upper and lower lips with white and black dots; supratympanic fold pale brown; iris golden brown; dorsal parts of arms and legs with dark brown crossbars that align; crotch with a distinct black patch bordering large creamy white plaque below the black patch near the groin; dorsal thigh beige with one brown crossbar when leg is bent in resting position; ventral surface body and beige, and area around vent with small black spots; discs of fingers and toes yellow.

##### Coloration in alcohol.

**Figure 5.**
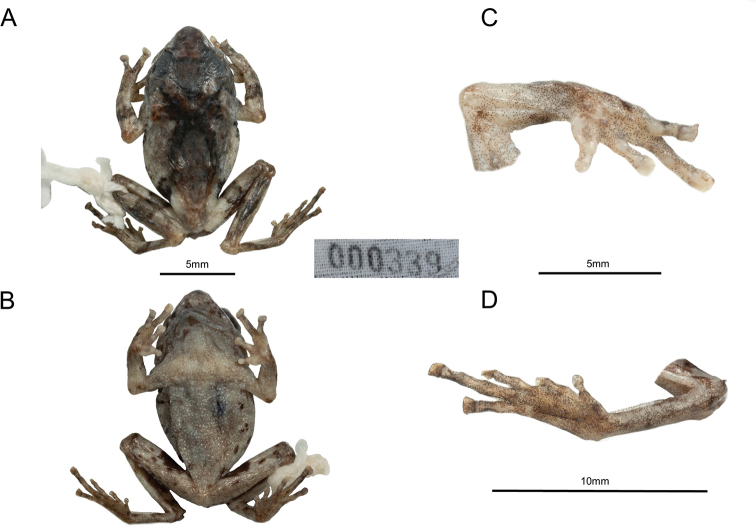
Holotype (GXNU 000339) of *Raorchestesmalipoensis* sp. nov. in preservative, showing **A** dorsal view **B** ventral view **C** ventral view of hand **D** ventral view of foot.

After preservation in alcohol, the general pattern did not change. Dorsal color changed to grayish brown, the blotches or spots blackish brown, discs on the fingers become pale gray similar to the body color, ventral side become whiter (Fig. 5).

##### Etymology.

The specific epithet is named for the type locality, Malipo County, Yunnan Province, China. We suggest “Malipo Bush Frog” as its English common name, and “Ma Li Po Guan Shu Wa (麻栗坡灌树蛙)” as its Chinese common name.

##### Distribution.

Currently known from the type locality, Malipo County (Fig. 1), Yunnan Province, China and Pac Ban, Tuyen Quang, in north of Vietnam.

##### Variation.

The measurements are given in Table 1. GXNU 000338 has large black spots on dorsal side and GXNU000342 has distinctly darker ground color on dorsal side.

##### Comparisons.

Rather than comparing *R.malipoensis* sp. nov. to all known *Raorchestes*, we focus on our morphological comparison with phylogenetically closely related taxa and species without genetic data in adjacent countries (Table 5).

**Table 5.** Comparison of *R.malipoensis* sp. nov. with phylogenetically closely related taxa or those with no genetic data in surrounding countries. “–” means unknown.

Species	*R.malipoensis* sp. nov.	*R.menglaensis*	*R.parvulus*	*R.dulongensis*	*R.hillisi*	*R.huanglianshan*	*R.cangyuanensis*	*R.ghatei*	*R.rezakhani*	*R.annandalii*	*R.bombayensis*	*R.tuberohumerus*	*R.longchuanensis*	*R.andersoni*
SVL of adult males (in mm)	14.6–17.7, *n* = 7	16.6–21.6, *n* = 14	–	15.0–19.0, *n* = 3	14.5–17.7, *n* = 3	17.0–19.6, *n* = 11	16.1–19.0 mm, *n* = 3	19.1–25.5, *n* = 9	18.8–19.0 mm, *n* = 4	–	30 mm, *n* = –	17.4–18.2 mm, *n* = 3	21.4–23.9 mm, *n* = 5	13.5–24.0 mm, *n* = 2
SVL of adult females (in mm)	18.3–19.3, *n* = 4	18.9–20.5, *n* = 2	23.6, *n* = 1	–	17.5, *n* = 1	21.5, *n* = 1	–	15.4–29.8, *n* = 13	–	17.0 mm, *n* = 1		–	–	–
SVL of adult (in mm)	14.6–19.3, *n* = 11	16.6–21.6, *n* = 18	23.6, *n* = 1	15.0–19.0, *n* = 3	14.5–17.7, *n* = 4	17.0–21.5, *n* = 4	16.1–19.0 mm, *n* = 3	15.4–29.8, *n* = 22	18.8–19.0 mm, *n* = 4	17.0 mm, *n* = 1	30 mm, *n* = –	17.4–18.2 mm, *n* = 3	21.4–23.9 mm, *n* = 5	13.5–24.0 mm, *n* = 2
IOS/EHD	IOS > EHD, or IOS = EHD	IOS > EHD	IOS < EHD	IOS < EHD	IOS < EHD	IOS > EHD, or IOS=EHD	IOS < EHD	–	IOS < EHD	IOS > EHD, or IOS = EHD	–	IOS > EHD, or IOS = EHD	IOS > EHD	IOS < EHD
HDW/HDL	HDW > HDL	HDW < HDL	HDW > HDL	HDW < HDL	HDW < HDL	HDW > HDL	HDW > HDL	HDW > HDL	HDW > HDL	HDW < HDL	–	HDW > HDL	HDW ≈ HDL	HDW > HDL
Tympanum	Distinct	Indistinct	Distinct	Distinct	Distinct	Distinct	Indistinct	Indistinct	Indistinct	Distinct	Indistinct	Indistinct	Distinct	Distinct
Nuptial pad	Small and milky white	white nuptial pad	–	Absent	Present	Present	Reddish nuptial pad	Absent	Absent	–	–	–	Present	–
Toe web	I 2 – 2 II 2 – 2 III 2 – 3 IV 3 – 2 V	II 1 – 2 III 1 – 2^1/2^ IV 2^1/2^ – 1 V	Webbing present, medium	Rudimentary web	II 1 – 2 III 1–2^1/2^ IV 2^1/2^ – 1 V	II 1 – 2 III 1 – 2 – IV 2 – 1 V	Rudimentary web	I 2 – 2 II 2 – 2½ III 2 – 3 IV 2½ – 2 V	I2 – 2 II 1¾ – 2 III 1½ – 3 IV 2¾ – 2 V	Rudimentary web	1/3 webbing	Rudimentary web	1/4 webbing	1/3 webbing
Lateral dermal fringe	Present	Absent	–	–	Present	Absent	Present	Present	Absent	Present	–	–	–	–
Disc color	Yellow	Not orange in life	–	Greyish or orange	–	Orange	Orange	–	Reddish or whitish	–	–	–	Reddish, orange, or whitish	orange
Inner metacarpal tubercle	Indistinct	Present	Present	Present	Indistinct	Indistinct	–	–	Absent	Present	–	–	Present	Present
Outer metacarpal tubercle	Indistinct	Present	Present	Present	Indistinct	Indistinct	–	–	Absent	Present	–	–	Present	Present
Inner metatarsal tubercle	Round	Present	Present	Round	Round	Round	Round	Round	Absent	Absent	–	Present	Present	Present
Outer metatarsal tubercle	Absent	Present	Absent	Absent	Absent	Absent	Absent	Absent	Absent	Absent	–	Absent	Absent	Absent
Relative toe lengths	I < II < V < III < IV	III ≈ V, or V > III	I < II < V < III < IV	I < II < V < III < IV	I<II<III<V<IV	I<II<III<V<IV	I<II<V<III<IV	I<II<V=III<IV	I < II < V < III < IV	I < II < V = III < IV	–	I < II ≤ V < III < IV	III ≈ V	I < II < III = V < IV
Range	Malipo, Yunnan, China and the north of Vietnam	Mengla, Yunnan, China	Indochina Peninsula and peninsular Malaysia	Gongshan, Yunnan, China	Menghai, Yunnan, China	Lvchun, Yunnan, China	Cangyuan, Yunnan, China	Western Ghats, India	Northeastern Bangladesh	Himalayas and northeastern India	Western Ghats, India	Western Ghats, India	Yunnan, China and Lai Chau,Vietnam	India, North Myanmar, Tibet and Yunnan, China

The new species differs from *R.menglaensis* by 1) tubercles absent along the outer side of the forearm and foot; (2) head wider than long; (3) tympanum distinct (TD 1.1–1.6 mm, *n* = 11); (4) webbing formula (I 2 – 2 II 2 – 2 III 2 – 3 IV 3 – 2 V); (5) lateral dermal fringe present (6) inner and outer metacarpal tubercle indistinct; (7) outer metatarsal tubercle absent; and (8) relative toe lengths: I < II < V < III < IV (vs. a series of tubercles along the outer side of the forearm and foot; head length and head width are approximately the same; tympanum indistinct; webbing formula (II 1 – 2 III 1 – 2^1/2^ IV 2^1/2^–1 V); lateral dermal fringe present; inner and outer metatarsal tubercle present; outer metatarsal tubercle present; relative toe lengths: III ≈ V, or V > III).

The new species differs from *R.parvulus* by (1) smaller female body size (females 18.3–19.3 mm, *n* = 4); (2) interorbital distance larger than eye horizontal diameter; and (3) inner and outer metacarpal tubercle indistinct; (vs. female 23.6 mm, *n* = 1; interorbital distance smaller than eye horizontal diameter; inner and outer metacarpal tubercle present).

The new species differs from *R.dulongensis* by (1) head wider than long; (2) interorbital distance larger than eye horizontal diameter; (3) nuptial pad present; (4) yellow disc; and (5) inner and outer metacarpal tubercle indistinct (vs. head smaller than long; interorbital distance smaller than eye horizontal diameter; nuptial pad absent; greyish or orange disc; inner and outer metacarpal tubercle indistinct present).

The new species differs from *R.hillisi* by (1) larger female body size (females 18.3–19.3 mm, *n* = 4); (2) head wider than long; (3) interorbital distance larger than eye horizontal diameter; (4) webbing formula (I 2 – 2 II 2 – 2 III 2 – 3 IV 3 – 2 V) ; and (5) and relative toe lengths: I < II < V < III < IV (vs. female 17.5 mm, *n* = 1; head longer than wider; interorbital distance smaller than eye horizontal diameter; webbing formula (II 1–2 III 1–2^1/2^ IV 2^1/2^–1 V); relative toe lengths: I < II < III < V <IV).

The new species differs from *R.huanglianshan* by (1) smaller female body size (females18.3–19.3 mm, *n* = 4); (2) lateral dermal fringe present; (3) yellow disc; (4) webbing formula (II 2 – 2 II 2 – 2 III 2 – 3 IV 3 – 2 V); and (5) relative toe lengths: I < II < V < III < IV (vs. female 21.5 mm, *n* = 1; lateral dermal fringe absent; orange disc; fingers and toes lacking lateral dermal fringe; webbing formula (II 1–2 III 1 – 2 – IV 2 – 1 V); relative toe lengths: I < II < III < V < IV).

The new species differs from *R.cangyuanensis* by (1) interorbital distance larger than eye horizontal diameter; (2) nuptial pad small and milky white; and (3) yellow discs (vs. interorbital distance smaller than eye horizontal diameter; reddish nuptial pad at the base of first finger; orange disc).

The new species differs from *R.ghatei* by (1) smaller body size (males 14.6–17.7 mm, *n* = 7; females18.3–19.3 mm, *n* = 4); (2) tympanum distinct (TD 1.1–1.6 mm, *n* = 11); (3) nuptial pad present; (4) webbing formula (I 2 – 2 II 2 – 2 III 2 – 3 IV 3 – 2 V); and (5) relative toe lengths: I < II < V < III < IV (vs. males 19.1–25.5 mm, *n* = 9; females 15.4–29.8 mm, *n* = 13; tympanum indistinct; nuptial pad absent; webbing formula (I 2 – 2 II 2 – 2½ III 2– 3 IV 2½ – 2 V); relative toe lengths: I < II < V = III < IV).

The new species differs from *R.rezakhani* by (1) smaller male body size (males 14.6–17.7 mm, *n* = 7); (2) interorbital distance larger than eye horizontal diameter; (3) tympanum distinct (TD 1.1–1.6 mm, *n* = 11); (4) nuptial pad present; (5) lateral dermal fringe present; (6) yellow disc; (7) inner and outer metacarpal tubercle indistinct; (8) inner metatarsal tubercle round; and (9) webbing formula (I 2 – 2 II 2 – 2 III 2 – 3 IV 3 – 2 V) (vs. males 18.8–19.0 mm; interorbital distance smaller than eye horizontal diameter; tympanum indistinct; nuptial pad absent; lateral dermal fringe absent; reddish or whitish; inner and outer metacarpal tubercle absent; inner metatarsal tubercle absent; webbing formula (I2 – 2 II 1¾ – 2 III 1½ – 3 IV 2¾ – 2 V).

The new species differs from *R.annandalii* by (1) head wider than long; and (2) relative toe lengths: I < II < V < III < IV (vs. head longer than wide; relative toe lengths: I < II < V = III < IV).

The new species differs from *R.bombayensis* by (1) smaller body size (males 14.6–17.7 mm, *n* = 7; females 18.3–19.3 mm, *n* = 4); (2) tympanum distinct (TD 1.1–1.6 mm, *n* = 11); and (3) webbing formula (I 2 – 2 II 2 – 2 III 2 – 3 IV 3 – 2 V) (vs. 30 mm, *n* = 1; tympanum indistinct; 1/3 webbing between toes).

The new species differs from *R.tuberohumerus* by (1) tympanum distinct (TD 1.1–1.6 mm, *n* = 11); and (2) relative toe lengths: I < II < V < III < IV (vs. tympanum indistinct; relative toe lengths: I < II ≤ V < III < IV).

The new species differs from *R.longchuanensis* by (1) smaller male body size (males 14.6–17.7 mm, *n* = 7); (2) webbing formula (I 2 – 2 II 2 – 2 III 2 – 3 IV 3 – 2 V); and (3) yellow disc (vs. males 21.4–23.9 mm, *n* = 5; 1/4 webbing between toes; reddish, orange, or whitish disc).

The new species differs from *R.andersoni* by (1) interorbital distance larger than eye horizontal diameter; (2) webbing formula (I 2 – 2 II 2 – 2 III 2 – 3 IV 3 – 2 V); (3) yellow disc; and (4) relative toe lengths: I < II < V < III < IV (vs. interorbital distance smaller than eye horizontal diameter; 1/3 webbing between toes; orange disc; relative toe lengths: I < II < III = V < IV).

## ﻿Discussion

Recently, Poyarkov et al. (2021) placed *Philautusgryllus* in the genus *Kurixalus* based on unpublished molecular evidence and a study of type materials. In this study, the sample previously identified as *R.gryllus* from northern Vietnam (voucher number: ROM 30288) nests in the clade of *R.malipoensis* sp. nov. without distinct genetic divergence (Table 4), indicating that they are likely conspecific (Table 4). Morphologically, *Raorchestesmalipoensis* sp. nov. is obviously distinguishable from *K.gryllus* as described by Smith (1924; Table 6) by (1) smaller body size 14.6–19.3 mm, *n* = 11; (2) tympanum distinct (TD 1.1–1.6 mm, *n* = 11); (3) webbing formula (I 2 – 2 II 2 – 2 III 2 – 3 IV 3 – 2 V); (4) no webbing between fingers; (5) outer metatarsal tubercle absent (vs. 25.0–27.0 mm, *n* = 3; tympanum distinct; toes a little more than half webbed; fingers free except for a rudiment of a web between the two outer; outer metatarsal tubercle separated for approximately two-thirds of their length). Therefore, we consider that *Raorchestesmalipoensis* sp. nov. is not conspecific with *K.gryllus* and the record of *R.gryllus* (ROM 30288) from northern Vietnam should be revised to *R.malipoensis* sp. nov. We also suggest that the taxonomic status of other records of *R.gryllus* from Vietnam and Laos need further examinations.

**Table 6.** Morphological comparison between *Raorchestesmalipoensis* sp. nov. and *Kurixalusgryllus* (Smith, 1924).

Character	Species	
	*Raorchestesmalipoensis* sp. nov. (n = 11)	*Kurixalusgryllus* (n = 3)
SVL	14.6–19.3 mm	25.0–27.0 mm,
HL	5.2–7.9 mm	8.0–9.5 mm
HW	5.5–8.2 mm	10.0–11.0 mm
EHD	2.1–2.8 mm	3.0–3.5 mm
SL	1.8–2.9 mm	4–4.5 mm
HAL	4.2–5.6 mm	7.5–8.5 mm
TBL	7.5–9.2 mm	12–13 mm
TD	1.1–1.6 mm *n* = 11	tympanum indistinct
Tubercles along forearm and foot	absent	present
Web of toes	I 2 – 2 II 2 – 2 III 2 – 3 IV 3 – 2 V	toes a little more than half webbed
Web of fingers	no webbing between fingers	fingers free except for a rudiment of a web between the two outer fingers
Metatarsal tubercle	inner metatarsal tubercle rounded, outer metatarsal tubercle absent	a small inner metatarsal tubercle
Coloration	dorsal surface beige, with pale brown and dark brown spots, an individual having large black spots on its body surface	dorsal color with pale or dark brown, green, yellow, or grey, many individuals had a bright green patch on the snout, and patches of similar color on the knees and round the vent

In recent years, many new species have been found along the border between China and Vietnam, such as *Odorranageminata* (Bain et al., 2009), *Tylototritonziegleri* (Nishikawa et al., 2013), *Leptobrachellafeii* (Chen et al., 2020), *Amolopsshihaitaoi* (Wang et al., 2022), and *Thelodermahekouense* (Du et al., 2022). Tropical montane forests in the border region between China and Vietnam are known to harbor a high level of species richness and local endemism (Sterling et al. 2006). One of the main reasons assumed to be responsible for this richness is the greater environmental heterogeneity observed in the montane regions as opposed to the lowland regions, allowing for a larger number of habitats to be occupied by species (Keller et al. 2009). It is expected that more new species from this region would be discovered, and further studies are required to accurately determine the species richness of tree frogs in China-Vietnam border region. Due to historical reasons, herpetological surveys of this region had been scarce, but considering the biogeographical interest of the region it is important to facilitate collaborative research to comprehensively understand herpetofaunal diversity, community composition, and species range limits around the region in order to better protect them and their environment in the face of global warming and habitat destruction.

## Supplementary Material

XML Treatment for Raorchestes malipoensis

## References

[B1] Al-Razi H Maria M Hasan S Muzaffar SB (2020a) A new species of cryptic Bush frog (Anura, Rhacophoridae, *Raorchestes*) from northeastern Bangladesh. ZooKeys 927: 127–151. 10.3897/zookeys.927.48733 32341678 PMC7180169

[B2] Al-Razi H Maria M Hasan S Muzaffar SB (2020b) First record of *Raorchesteslongchuanensis* Yang & Li, 1978 (Anura: Rhacophoridae) from northeastern Bangladesh suggests wide habitat tolerance. Amphibian & Reptile Conservation 14(1[: e225]): 119–131.

[B3] Anderson J (1878) Anatomical and zoological researches: Comprising an account of the zoological results of the two expeditions to Western Yunnan in 1868 and 1875. Vol 1. Bernard Quaritch, London, 703–860 + 969–975. [index] 10.5962/bhl.title.50434

[B4] Bain RH Stuart BL Nguyen TQ Che J Rao DQ (2009) A new *Odorrana* (Amphibia: Ranidae) from Vietnam and China. Copeia 2009(2): 348–362. 10.1643/CH-07-195

[B5] Biju SD Bossuyt F (2009) Systematics and phylogeny of *Philautus* Gistel, 1848 (Anura, Rhacophoridae) in the Western Ghats of India, with descriptions of 12 new species. Zoological Journal of the Linnean Society 9(155): 374–444. 10.1111/j.1096-3642.2008.00466.x

[B6] Biju SD Shouche Y Dubois A Dutta SK Bossuyt F (2010) A ground-dwelling rhacophorid frog from the highest mountain peak of the Western Ghats of India. Current Science 98(8): 1119–1125.

[B7] Bossuyt F Dubois A (2001) A review of the frog genus *Philautus* Gistel, 1848 (Amphibia, Anura, Ranidae, Rhacoporinae). Zeylonica 6: 1–112.

[B8] Boulenger GA (1893) Concluding report on the reptiles and batrachians obtained in Burma by Signor L. Fea dealing with the collection made in Pegu and the Karin Hills in 1887–88. Annali del Museo Civico di Storia Naturale di Genova Serie 2(13): 304–347.

[B9] Chan KO Grismer LL Brown RM (2018) Comprehensive multi-locus phylogeny of Old World tree frogs (Anura: Rhacophoridae) reveals taxonomic uncertainties and potential cases of over-and underestimation of species diversity. Molecular Phylogenetics and Evolution 127: 1010–1019. 10.1016/j.ympev.2018.07.005 30030179

[B10] Che J Jiang K Yan F Zhang YP (2020) Amphibians and Reptiles in Tibet-Diversity and Evolution. Science Press, Beijing, 803 pp. [in Chinese]

[B11] Chen JM Xu K Poyarkov NA Wang K Yuan ZY Hou M Suwannapoom C Wang J Che J (2020) How little is known about “the little brown frogs”: description of three new species of the genus *Leptobrachella* (Anura: Megophryidae) from Yunnan Province, China. Zoological Research 41: 1–22. 10.24272/j.issn.2095-8137.2020.036 32323508 PMC7231475

[B12] Darriba D Taboada GL Doallo R Posada D (2012) jModelTest 2: More models, new heuristics and parallel computing. Nature Methods 9(8): 772. 10.1038/nmeth.2109 PMC4594756 22847109

[B13] Fei L Hu SQ Ye CY Huang YZ (2009) Fauna Sinica. Amphibia Vol. 2 Anura. Science Press, Beijing, 957 pp. [In Chinese]

[B14] Frost DR (2021) Amphibian Species of the World: an Online Reference. Version 6.1. Electronic Database. American Museum of Natural History, New York, USA. http://research.amnh.org/herpetology/amphibia/index.html [accessed 12 September 2021]

[B15] Jiang K Ren JL Wang J Guo JF Wang Z Liu YH Jiang DC Li JT (2020) Taxonomic revision of *Raorchestesmenglaensis* (Kou, 1990) (Amphibia: Anura), with descriptions of two new species from Yunnan, China. Asian Herpetological Research 11(4): 263–281. 10.16373/j.cnki.ahr.200018

[B16] Keller A Rödel MO Linsenmair KD Grafe TU (2009) The importance of environmental heterogeneity for species diversity and assemblage structure in Bornean stream frogs. Journal of Animal Ecology 78(2): 305–314. 10.1111/j.1365-2656.2008.01457.x 18671805

[B17] Kou ZT (1990) A new species of genus *Philautus* (Amphibia: Rhacophoridae) from Yunnan, China. In: From Water onto Land. China Forestry Press, Beijing, 210–212. [In Chinese]

[B18] Kuramoto M Joshy SH (2003) Two new species of the genus *Philautus* (Anura: Rhacophoridae) from the Western Ghats, southwestern India. Current Herpetology 22(2): 51–60. 10.5358/hsj.22.51

[B19] Li JT Che J Murphy RW Zhao H Zhao EM Rao DQ Zhang YP (2009) New insights to the molecular phylogenetics and generic assessment in the Rhacophoridae (Amphibia: Anura) based on five nuclear and three mitochondrial genes, with comments on the evolution of reproduction. Molecular Phylogenetics and Evolution 53(2): 509–522. 10.1016/j.ympev.2009.06.023 19616637

[B20] Meegaskumbura M Bossuyt F Pethiyagoda R Manamendra-Arachchi K Bahir M Milinkovitch MC Schneider CJ (2002) Sri Lanka: An amphibian hot spot. Science 298(5592): 379. 10.1126/science.298.5592.379 12376694

[B21] Nishikawa K Matsui M Nguyen TT (2013) A new species of *Tylototriton* from northern Vietnam (Amphibia: Urodela: Salamandridae). Current Herpetology 32(1): 34–49. 10.5358/hsj.32.34

[B22] Padhye AD Sayyed A Jadhav A Dahanukar N (2013) *Raorchestesghatei*, a new species of shrub frog (Anura:Rhacophoridae) from the Western Ghats of Maharashtra, India. Journal of Threatened Taxa 5(15): 4913–4931. 10.11609/JoTT.o3702.4913-31

[B23] Palumbi SR Martin AP Romano SL McMillan WO Stice L Grabowski G (1991) The Simple Fool’s Guide to PCR. Special Publications. Department of Zoology, University of Hawaii, Honolulu, Hawaii, USA, 94 pp.

[B24] Posada D (2008) jModelTest: Phylogenetic model averaging. Molecular Biology and Evolution 25(7): 1253–1256. 10.1093/molbev/msn083 18397919

[B25] Poyarkov NA Nguyen TV Popov ES Geissler P Pawangkhanant P Thy N Suwannapoom C Orlov NL (2021) Recent progress in taxonomic studies, biogeographic analysis, and revised checklist of amphibians in Indochina. Russian Journal of Herpetology 28(3): 1–110. 10.30906/1026-2296-2021-28-3A-1-110

[B26] Priti H Roshmi RS Ramya B Sudhira HS Ravikanth G Aravind NA Gururaja KV (2016) Integrative taxonomic approach for describing a new cryptic species of Bush Frog (*Raorchestes*: Anura: Rhacophoridae) from the Western Ghats, India. PLoS ONE 11(3): e0149382. 10.1371/journal.pone.0149382 PMC4774957 26934213

[B27] Rambaut A Drummond AJ (2009) Tracer. Version 1.5. http://tree.bio.ed.ac.uk/software/tracer/ [accessed 3 September 2022]

[B28] Ronquist F Teslenko M van der Mark P Ayres DL Darling A Höhna S Larget B Liu L Suchard MA Huelsenbeck JP (2012) MrBayes 3.2: Efficient Bayesian phylogenetic inference and model choice across a large mod space. Systematic Biology 61(3): 539–542. 10.1093/sysbio/sys029 22357727 PMC3329765

[B29] Seshadri KS Gururaja KV Aravind NA (2012) A new species of *Raorchestes* (Amphibia: Anura: Rhacophoridae) from mid-elevation evergreen forests of the south Western Ghats, India. Zootaxa 3410(1): 19–34. 10.11646/zootaxa.3410.1.2

[B30] Smith MA (1924) New tree-frogs from Indo-China and the Malay Peninsula. 94. Proceedings of the Zoological Society of London, London, 225–234. 10.1111/j.1096-3642.1924.tb01499.x

[B31] Stamatakis A (2014) RAxML version 8: A tool for phylogenetic analysis and post-analysis of large phylogenies. Bioinformatics 30(9): 1312–1313. 10.1093/bioinformatics/btu033 24451623 PMC3998144

[B32] Sterling EJ Hurley MM Le MD (2006) Vietnam: a natural history. Yale University, New Haven, 448 pp.

[B33] Subramanian KA Dinesh KP Radhakrishnan C (2013) Atlas of endemic amphibians of Western Ghats. Zoological Survey of India, Kolkata, 246 pp.

[B34] Tamura K Stecher G Peterson D Filipski A Kumar S (2013) MEGA6: Molecular evolutionary genetics analysis version 6.0. Molecular Biology and Evolution 30(12): 2725–2729. 10.1093/molbev/mst197 24132122 PMC3840312

[B35] Vijayakumar SP Dinesh KP Prabhu MV Shanker K (2014) Lineage delimitation and description of nine new species of bush frogs (Anura: *Raorchestes*, Rhacophoridae) from the Western Ghats Escarpment. Zootaxa 3893(4): 451–488. 10.11646/zootaxa.3893.4.1 25544534

[B36] Wu YH Suwannapoom C Xu K Chen JM Jin JQ Chen HM Murphy RW Che J (2019) A new species of the genus *Raorchestes* (Anura: Rhacophoridae) from Yunnan Province, China. Zoological Research 40(6): 558–563. 10.24272/j.issn.2095-8137.2019.066 31631588 PMC6822928

[B37] Wu YH Liu XL Gao W Wang YF Li YC Zhou WW Yuan ZY Che J (2021) Description of a new species of Bush frog (Anura: Rhacophoridae: *Raorchestes*) from northwestern Yunnan, China. Zootaxa 4941(2): 239–258. 10.11646/zootaxa.4941.2.5 33756941

[B38] Yan F Liu XL Zhang YP Yuan ZY (2021) Direct development of the bush frog *Raorchesteslongchuanensis* (Yang & Li, 1978) under laboratory conditions in Southern China. Journal of Natural History 55(1–2): 125–132. 10.1080/00222933.2021.1895349

[B39] Yang DT Li SM (1978) In: Yang DT, Su CY, Li SM (Eds) Amphibians and Reptiles of Gaoligongshan, Kunming 8: 37–38. [In Chinese]

